# Pentoxifylline inhibits the fibrogenic activity of pleural effusions and transforming growth factor-β

**DOI:** 10.1080/09629359791811

**Published:** 1997-04

**Authors:** P. Entzian, M. Schlaak, U. Seitzer, Y. Acil, M. Ernst, P. Zabel

**Affiliations:** 1 Medizinische Klinik Forschungszentrum Borstel Parkallee 35 Borstel 23845 Germany; 2 Institutsbereich Immunologie und Zellbiologie Forschungszentrum Borstel Germany; 3 Institut für Medizinische Molekularbiologie Medizinische Universitaät Lübeck Germany

## Abstract

Physiopathology of organ fibrosis is far from being completely understood, and the efficacy of the available therapeutic strategies is disappointing. We chose pleural disease for further studies and addressed the questions of which cytokines are relevant in pleural fibrosis and which drugs might interrupt its development. We screened pleural effusions for mediators thought to interfere with fibrogenesis (transforming growth factor-β (TGF-β), tumour necrosis factor α (TNFα), soluble TNF-receptor p55 (sTNF-R)) and correlated the results with patient clinical outcome in terms of extent of pleural thickenings. We found pleural thickenings correlated with TGF-β (p < 0.005) whereas no correlations could be observed with TNFα and sTNF-R. Further, we were interested in finding out how TGF-β effects on fibroblast growth could be modulated. We found that pentoxifylline is able to inhibit both fibroblast proliferation and collagen synthesis independently of the stimulus. We conclude that, judging from *in vitro* studies, pentoxifylline might offer a new approach in the therapy of pleural as well as pulmonary fibrosis.


Mediators of Inflammation, 6, 119–126 (1997)

### Pentoxifylline inhibits the fibrogenic activity of pleural effusions and transforming growth factor-β

P. Entzian,[1,CA] M. Schlaak,[1] U. Seitzer,[2] Y. Acil,[3] M. Ernst[2] and P. Zabel[1]

[1]Medizinische Klinik, Forschungszentrum Borstel, Parkallee 35, 23845 Borstel, Germany; [2]Institutsbereich Immunologie und Zellbiologie, Forschungszentrum Borstel, Germany; [3]Institut für Medizinische Molekularbiologie, Medizinische Universität Lübeck, Germany

[CA]Corresponding Author
Tel: (+49) 4537 188 0
Fax: (+49) 4537 188 313

**PHYSIOPATHOLOGY of organ fibrosis is far from being completely understood, and the efficacy of the available therapeutic strategies is disappointing. We chose pleural disease for further studies and addressed the questions of which cytokines are relevant in pleural fibrosis and which drugs might interrupt its development. We screened pleural effusions for mediators thought to interfere with fibrogenesis (transforming growth factor-β (TGF-β), tumour necrosis factor α (TNFα), soluble TNF-receptor p55 (sTNF-R)) and correlated the results with patient clinical outcome in terms of extent of pleural thickenings. We found pleural thickenings correlated with TGF-β ($P < 0.005$) whereas no correlations could be observed with TNFα and sTNF-R. Further, we were interested in finding out how TGF-β effects on fibroblast growth could be modulated. We found that pentoxifylline is able to inhibit both fibroblast proliferation and collagen synthesis independently of the stimulus. We conclude that, judging from *in vitro* studies, pentoxifylline might offer a new approach in the therapy of pleural as well as pulmonary fibrosis.**

**Key words:** Collagen, Fibroblast, Fibrosis, Pentoxifylline, Pleural effusion, Therapy, Transforming growth factor β, Tumour necrosis factor α

### Introduction

The normal fibrotic response to lung tissue injury is finely controlled. Despite multifactorial origins, mesenchymal cells migrate to the site of injury, proliferate and subsequently synthesize extracellular matrix components.[1] Neither the factors that contribute to limited matrix production in wound healing nor those that control excess or sustained matrix production in fibrosis have been fully characterized. Cytokines have been considered highly important in the development of pulmonary fibrosis (e.g. platelet derived growth factor (PDGF), tumour necrosis factor α (TNFα), transforming growth factor β (TGF-β)).[2–5] A therapeutic breakthrough has, however, not been forthcoming. Corticosteroids are still the first-line therapeutics in pulmonary fibrosis, even though they improve the course of disease in only about 25% of cases, and that to an often disappointing extent.[6] Alternative therapeutic regimen are either toxic, not convincingly effective or have not yet been subjected to sufficient study.[6]

We chose pleural disease for further studies on the physiopathology of fibrosis and therapeutic intervention strategies. Mesothelial cells of the pleura, being highly susceptible to harmful events,[7] exfoliate and uncover the submesothelial connective tissue. An exudative inflammatory reaction results with the accumulation of fluids in the pleural space.[8] These fluids contain a variety of substances with the potential to influence cell growth.[9,10] Pleural effusions are most intimately in contact with extracellular matrix producing submesothelial connective tissue, so we hypothesized they might reflect the complex mechanisms of inflammation, wound healing, and development of pleural fibrosis. We addressed the question of whether mediators considered to be causally involved in fibrosis of the lungs by either stimulating matrix synthesis (TGF-β[11]) or inhibiting cytokines or matrix production (soluble TNF-receptor p55 (sTNF-R),[12] are also physiopathologically relevant in pleural fibrosis.

Having worked out comparative mediator profiles and clinical patient outcomes, we investigated the effect of the cytokine found to be relevant (TGF-β) and of pleural effusions, a material containing a multiplicity of mediators, on *in vitro* fibroblast cultures in order to search for alternatives to steroid treatment. Screening various drugs, we chose the xanthine derivative

© 1997 Rapid Science Publishers pentoxifylline (POF) for comparative studies. Pentoxifylline is a methylxanthine initially prescribed in the therapy of peripheral vascular disease[13] which has been shown to interact with several cell types including fibroblasts.[14] Our rationale for this choice included the growing set of data indicating that the intracellular concentration of cyclic adenosine monophosphate (cAMP) influences fibroblast activity.[15,16] Pentoxifylline augments cAMP by inhibiting phosphodiesterase activity[16] and activating adenylate cyclase by inducing prostacyclin[17] and has been shown to inhibit fibroblast collagen, glycosaminoglycan and fibronectin synthesis and increase collagenase activity.[18] POF inhibits fibroblast proliferation stimulated by platelet-derived growth factor (PDGF), a further cytokine considered important in fibrosis.[19] The drug has also been shown to modulate inflammatory and immune reactions effectively (e.g. inhibition of leukocyte adhesion, aggregation, degranulation and superoxide release and monocyte TNF-α synthesis[16]). Thus we assumed that pentoxifylline's mechanism of action might constitute an effective therapeutic action in fibrosis.

In this paper, we provide data on (1) cytokine concentrations in pleural effusions (TGF-β, TNFα, sTNF-R); (2) pentoxifylline effects on (a) fibroblast cell proliferation, and (b) fibroblast collagen synthesis stimulated by pleural effusions and pure cytokines (TNFα, TGF-β). Concluded from these *in vitro* investigations, data is in favour of a pentoxifylline benefit in fibrosis.

### Materials and Methods

### Subjects

Unless otherwise indicated, proteins were analysed in pleural effusions from 49 consecutive patients suffering from non-carcinomatous pleurisy. Table 1 lists the pertinent clinical features and diagnoses. In all cases, thoracentesis or thoracoscopy was performed for diagnostic reasons. Only pleural fluids from the first diagnostic or therapeutic pleural intervention were used in this study. Routine analysis included the determination of specific weight, cell differentiation, total protein concentration and LDH concentration and subsequent classification as transudates (total protein in pleural fluid $< 30$ g/l, LDH-ratio pleural fluid:blood: $< 0.6$, or exudates (protein $> 30$ g/l, LDH-ratio $> 0.6$). Further analyses (cytomorphology, immunological markers, etc.) were done as appropriate. The fluids were centrifuged and the supernatant devoid of cellular components was stored at −80°C until further investigation.

In 24 of the subjects we were able to do follow-up studies. Pleural thickenings were measured using chest X-ray films or computed tomography (CT) scans of the thorax made 3–6 months after diagnosis during follow-up examinations and classified according to the international classification of radiographs of pneumoconioses (International Labor Office (ILO)).[20] The results were correlated with the concentrations of TGF-β, TNFα, and sTNF-R in the pleural effusions.

In seven of the patients, pleural effusions were co-cultivated *ex vivo* with a fibroblast cell culture. Details of these patients read as follows: pleural effusions occurred due to heart failure (patient 1, male, 39 years; patient 2, male, 61 years); due to neutrophil-rich parinfectious pleurisy (patient 3, male, 57 years); due to macrophage-rich parinfectious pleurisy (patient 4, male, 38 years; patient 5, male, 61 years), and due to tuberculosis (patient 6, male, 72 years; patient 7, male, 39 years).

### Assay of transforming growth factor-β

Measurement of TGF by bioassay (growth inhibition of a mink lung epithelial cell line) did not reveal reproducible results. Thus TGF-β 1 was determined by ELISA in which studies on recovery and reproducibility showed good results (Quantikinine, R&D Systems, MN, USA). Sample preparation included acidification using 1 mol/l acetic acid to split the molecule off the protein binding and subsequent dialysis using Visking membranes (Serva, Germany, exclusion limit: 8000–15000 dalton) against phosphate buffer solution (PBS, Gibco BRL, w/o calcium + magnesium). Thus, both the inactive, latent, and the active form of TGF-β are detected. The assay uses a sandwich enzyme immunoassay technique with a monoclonal antibody against TGF-β and a detection system using a polyclonal antibody conjugated to horseradish peroxidase. Optical density determined in a multiwell scanning spectrophotometer (ELISA-reader, Dynatech MR 5000) at a wavelength of 450 nm allowed calculation of TGF-β of the samples by comparison with a standard curve. Lower detection limit of the assay is at 5 pg/ml.

### Assay of tumour necrosis factor α

Sample preparation was not a prerequisite in this assay. The sandwich immunoassay (Medgenix Diagnostics, Belgium) consists of oligoclonal

**Table 1**. Pertinent clinical features of the patients investigated

| Diagnosis: effusions occurred due to: | n | Sex | Age (mean, range) | | No. of patients with pleural thickenings, ILO-grade 0 | 1a | 1b | 1c | 2a | 2b | 2c | 3a | 3b | 3c |
|---|---|---|---|---|---|---|---|---|---|---|---|---|---|---|
| Congestive heart failure | 17 | 7f<br>10m | 75<br>(56–89) | transudates | 15 | 2 | – | – | – | – | – | – | – | – |
| Parapneumonic | | | | | | | | | | | | | | |
| neutrophil-rich | 5 | 2f; 3m | 40 | exudates | | | | | | | | | | |
| macrophage-rich | 7 | 2f; 5m | (24–61) | exudates | 4 | 2 | – | – | 1 | – | – | 2 | 1 | 2 |
| Empyema | | | | | | | | | | | | | | |
| streptococcus pneumoniae: n = 1 | | | 68 | | | | | | | | | | | |
| not identified: n = 4 | 5 | 5m | (57–75) | exudates | – | 1 | – | 2 | 1 | – | – | – | – | 1 |
| Tuberculosis | 15 | 5f; 10m | 43<br>(26–74) | exudates | 1 | 5 | 1 | 2 | 2 | 1 | 1 | – | – | 2 |

Forty-nine patients were enrolled in the study. 3–6 months after diagnosis, pleural thickenings were found in follow-up examinations and classified 0–3c according to the international classification of radiographs of pneumoconioses (ILO). ILO 0 and ILO 1a were designated as *restitutio ad integrum. The primary site of disease being the pleura, neutrophil-rich fluids were designated empyema to differentiate them* from parapneumonic neutrophil effusions.

TNF-antibodies and an anti-TNF-antibody detection system conjugated to horseradish peroxidase. It detects total TNFα (i.e. monomeric, trimeric, receptor-bound, and non-bound TNF). Concentrations of TNF can be determined by comparing optical densities of samples with standard curves. No cross-reactivity has been reported with tumour necrosis factor β (TNF-β), interleukin-1 (IL-1), interleukin-2 (IL-2), interferon-α (IFN-α), interferon-β (IFN-β), or interferon-γ (IFN-γ) (product information Medgenix). Detection limit of the assay is at 3 pg/ml.

### Assay of soluble tumour necrosis factor receptor p55

This assay was a kind gift of Dr H. Gallati (Hoffmann-La Roche, Switzerland). Principle of the assay is a sandwich enzyme immunoassay technique consisting of a monoclonal TNF-receptor p55 antibody (mouse) and peroxidase-conjugated recombinant TNF-α. Concentrations of unknowns are calculated from optical densities determined at a wavelength of 450 nm using a ELISA-reader. Detection limit is 100 pg/ml. No cross-reactivity with IFN-α, IFN-γ, IL-1α, IL-1β, PDGF-AA, PDGF-AB, PDGF-BB has been reported (product information Hoffmann-La Roche).

### Assay of fibroblast proliferation

A human lung fibroblast cell line was obtained from the American Type Culture Collection (WI-38, derived from normal embryonic lung tissue of a caucasian female) and grown in Basal medium (Eagle) (BME) according to standard protocols. Only early passage cell cultures (days 15–35, split ratio 1:2 once weekly) were used in these experiments. Fibroblasts (WI-38) were seeded at subconfluent density of $0.1 \times 10^6$ cells/ml (medium BME, 10% FCS added) into 96-well flat bottom microtitre plates (0.1 ml per well, Greiner, Germany) and cultivated for 24 h. After a 1 h 'washout period' to reduce or remove FCS, BME medium without or with FCS 0.4%, and pleural effusions or cytokines (TGF-β1 at 3 ng/ml, human, expressed in *Escherichia coli*, Sigma Chemicals, USA; TNFα at 100 ng/ml, human, recombinant, Boehringer, Mannheim, Germany) as well as 1% antibiotics (penicillin 100 U/ml, streptomycin 100 U/ml, amphotericin 0.25 mg/ml)) were added. In respective experiments, medium was supplemented with pentoxifylline (Rentylin, Dr Rentschler, Laupheim, Germany, at 50 and 100 μg/ml). Medium was removed completely after 72 h, and 100 μl of the tetrazolium salt MTT (dimethylethimazol-diphenyltetrazolium bromide, Sigma, dissolved in PBS at 5 mg/ml) was added. MTT is converted by mitochondria of living cells to the blue coloured substance formazan, and the amount of formazan produced is proportional to the number of cells present.[21] The optical density was determined in an ELISA-reader at a wavelength of 550 nm.

Experiments were done six-fold; important results have been confirmed by counting the cells in a haemocytometer (Coulter counter, after preparation of the cell nuclei).

### Assay of collagen synthesis by in vitro cultured fibroblasts

Experiments using TGF-β or TNFα as stimulant were done in quadruplicate, and all others in duplicate. Pleural effusions of seven patients were investigated regarding stimulation of collagen synthesis and its inhibition by pentoxifylline (Rentylin, Dr Rentschler, Laupheim, Germany, at 50 µg/ml).

Principle of the assay is the incorporation of [$^3$H]-proline into proteins and its hydroxylation to [$^3$H]-hydroxyproline in collagenous proteins as outlined in Ref. 22. The method was adapted such that non-incorporated radioactivity was removed by ultrafiltration instead of dialysis. Briefly, fibroblasts (WI-38) were grown in 24-well flat bottom microtitre plates to visual confluency and incubated in the presence of 50 mg/ml ascorbate, pleural effusions at a final dilution of 1:5, or cytokines TNFα or TGF-β diluted in medium supplemented with 0.4% FCS (TNFα: human, recombinant, Boehringer, Mannheim, Germany; TGF-β1: human, expressed in *E. coli*, Sigma Chemicals, USA), and 10 mCi [$^3$H]-proline/ml medium (L-[2,3-3H]-proline, Dupont, USA). After 24 h, cell pellets as well as cell-free supernatants were harvested, freeze-thawed three times, pelleted by centrifugation, and the supernatant was washed four times and ultrafiltrated (centrifugal concentrators Microsep Filtron, Karlstein, Germany, molecular weight cutoff 10 K; aqua dest. supplemented with 0.3 ml/ml phenylmethansulphonfluorid (PMSF), proteinase-inhibitor, Roth, Germany). Subsequently, samples were resuspended in 6 N HCl (Merck), hydrolysed (110°C, 24 h), dried in a vacuum-desiccator, and the amounts of [$^3$H]-proline and [$^3$H]-hydroxyproline were determined by automated amino acid analysis. Collagen concentrations were calculated according to the method of Wiestner *et al.*[22] Collagen synthesis was expressed as percentage of collagen of generated total protein and as radio-labelled hydroxyproline per cell.

### Statistics

The data is provided in means ± SEM unless otherwise stated. Variance significance was calculated by means of the Mann–Whitney *U*-test, Spearman's rank correlation test and Wilcoxon's signed rank test. For comparisons, *P* values $< 0.05$ were adopted as significant.

### Results

### TGF-β, TNFα, and TNF-receptor p55 in pleural effusions

We found a significant positive correlation between the concentrations of TGF-β1 and the extent of pleural thickenings classified according to ILO as outlined in Fig. 1 (coefficient of correlation: 0.54; $P < 0.005$). Assuming a cutoff at TGF-β = 100 ng/ml and defining pleural thickenings ILO 0 and 1a to be a *restitutio ad integrum*, the sensitivity of TGF-β measurements to detect risk of pleural fibrosis was 75% and specificity was 80%

Table 2 provides data on TGF-β, TNFα, and

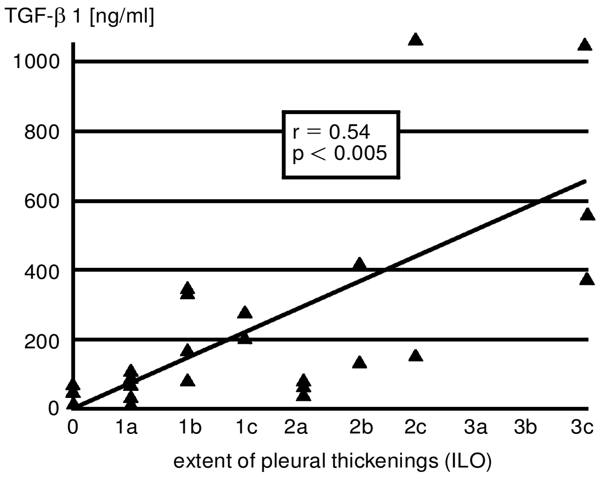

FIG. 1. TGF-β in pleural effusions due to infectious pleurisy. Total TGF-β isoform 1 (that is, both inactive protein-bound and active forms) was determined by ELISA and correlated with pleural thickening as determined by chest X-ray films or computed tomography scans, then classified as ILO 0–3c.

Table 2. Proteins in pleural effusions

| Diagnosis: effusions occurred due to: | *n* | *TGF*-β1 ng/g protein | TNFα ng/g protein | sTNF receptor p55 ng/g protein |
|---|---|---|---|---|
| Congestive heart failure | 17 | 3.95 ± 2.13 | 0.81 ± 1.43 | 2.55 ± 1.37 |
| Parapneumonic | | | | |
| neutrophil-rich | 5 | 2.38 ± 0.75 | 6.73 ± 7.77 | 0.78 ± 0.71 |
| macrophage-rich | 7 | 1.20 ± 0.30 | 0.54 ± 0.98 | 0.62 ± 0.55 |
| Empyema | 5 | 5.76 ± 7.51 | 3.37 ± 3.33 | 0.34 ± 0.23 |
| Tuberculosis | 15 | 4.84 ± 5.25 | 9.70 ± 11.84 | 0.98 ± 0.83 |

Transforming growth factor-β1 (TGF-β1), tumour necrosis factor α (TNFα), and soluble tumour necrosis factor receptor p55 (sTNF-R) were determined in non-carcinomatous pleural effusions. The primary site of disease being the pleura, neutrophil-rich fluids were designated empyema to differentiate them from parapneumonic neutrophil effusions. Data is given after normalization for total protein content in pleural effusions.

sTNF-R concentrations in relation to the various diagnoses in the study population. We determined that the protein amounts in the pleural transudates were lower than in the pleural exudates in general. However, results varied widely in all patients groups so that the differences did not reach significant levels.

### Effects of pentoxifylline on in vitro fibroblast proliferation stimulated by TGF-β or TNFα

TNFα, at 100 ng/ml, proved to be a stimulant of fibroblast proliferation (compared with the control: +38%), and addition of pentoxifylline nearly prevented the cells from proliferating altogether (Fig. 2).

TGF-β proved to be a very weak stimulant of fibroblast proliferation only when cells were already proliferating (i.e. already stimulated by 4% FCS (TGF-β at 3 ng/ml); stimulation was, compared with the control experiment, only 10%). There was no effect at all on quiescent (i.e. non-FCS stimulated) fibroblasts. Pentoxifylline reduced the TGF-β effect by ~50%, $P < 0.05$ as shown in Fig. 2.

Results have been confirmed by counting the cells in a haemocytometer.

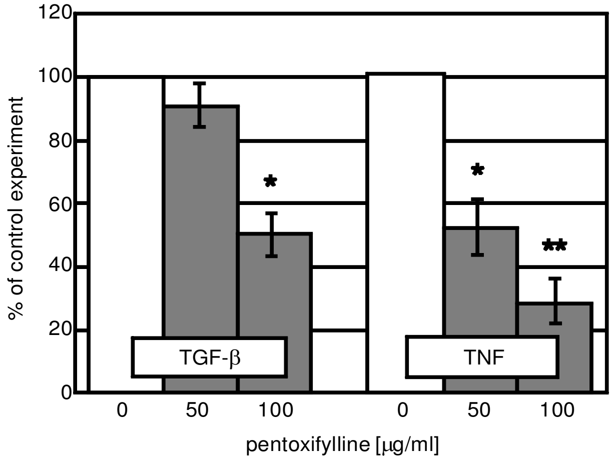

FIG. 2. Effects of pentoxifylline on in vitro fibroblast proliferation stimulated by TGF-β1 or TNFα. Fibroblasts (human lung fibroblast cell line WI-38) were seeded at subconfluent density and coincubated with TGF-β (3 ng/ml) or TNFα (100 ng/ml) and POF (50 or 100 µg/ml). Cell proliferation was measured after 72 h by MTT staining and calculated as % of inhibition by POF compared with control experiment results of stimulation by the respective cytokine alone. Both cytokines proved to be weak stimulants of fibroblast proliferation, which was significantly inhibited by addition of POF, 50 or 100 µg/ml. Increasing POF concentrations to above 100 µg/ml augmented inhibition of fibroblast proliferation. Data is provided in means ± SEM. Levels of significance: $^*P < 0.05$; $^{**}P < 0.025$. Important results have been confirmed by counting the cell nuclei in a haemocytometer (Coulter counter, after preparation of the cell nuclei).

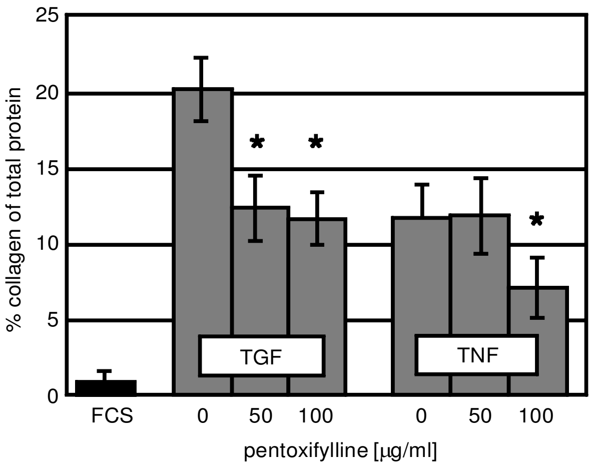

FIG. 3. Effects of pentoxifylline on in vitro fibroblast collagen synthesis stimulated by TGF-β1 (3 ng/ml) or TNFα (100 ng/ml). Fibroblasts (human lung fibroblast cell line WI-38) were investigated at confluent density. Collagen synthesis was determined by comparative measurement of incorporated [$^3$H]-proline and its hydroxylation product [$^3$H]-hydroxyproline and calculated as the collagen share in percentage of total protein formation. Synthesis was stimulated both by TNFα and TGF-β and was inhibited by concurrent addition of POF at 50 or 100 µg/ml to the culture medium. Data is provided in means ± SEM. Levels of significance: $^*P < 0.05$.

### Effects of pentoxifylline on in vitro fibroblast collagen synthesis stimulated by TGF-β or TNFα

Collagen synthesis was clearly stimulated by TGF-β. Addition of pentoxifylline, 50 or 100 µg/ml, inhibited collagen synthesis, reducing it by ~40% when stimulated with TGF-β at 3 ng/ml ($P < 0.05$). Stimulation with TGF-β at 20 ng/ml was not significantly inhibited by POF, although a slight decrease was observed (not shown). TNFα proved to be a weak stimulant of collagen synthesis which pentoxifylline was capable of inhibiting. The results were comparable when collagen synthesis was calculated per fibroblast.

### Effects of pleural effusions on in vitro fibroblast proliferation and collagen synthesis with and without addition of pentoxifylline

Pleural effusions in all patient groups stimulated *in vitro* fibroblast proliferation. Pentoxifylline inhibited proliferation significantly, $P < 0.025$ in experiments using pleural effusions as stimulant (see Fig. 4).

*In vitro* fibroblast collagen synthesis was also stimulated, varying between 3 and 12% of total protein synthesis. Pentoxifylline inhibited pleural effusion stimulated collagen synthesis significantly as shown in Fig. 5 ($P < 0.025$). Similar results were obtained when collagen

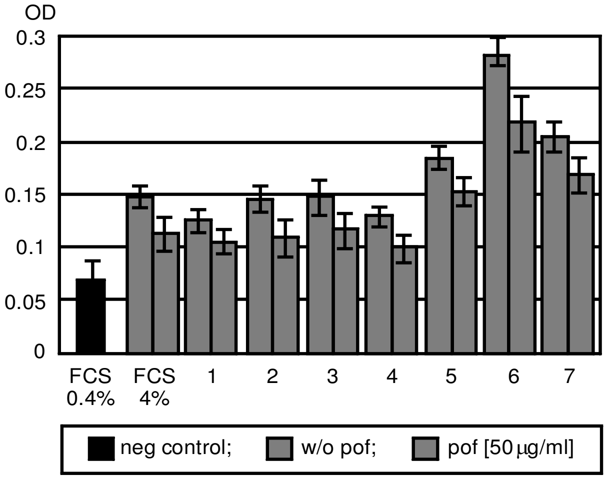

FIG. 4. Effect of pentoxifylline at 50 μg/ml on in vitro fibroblast proliferation, stimulated by fetal calf serum or by pleural effusions, with and without addition of pentoxifylline. Experiments were done six-fold. Effusions occurred due to: heart failure, patients 1 and 2; parinfectious pleurisy, patients 3, 4 and 5; tuberculosis, patients 6 and 7. Fibroblasts (human lung fibroblast cell line WI-38) were seeded at subconfluent density and coincubated with fetal calf serum (FCS) or pleural effusions and POF. Cell proliferation was measured after 72 h by MTT staining and determination of the optical density in an ELISA-reader. Results were confirmed by direct cell counting in a haemocytometer. It was proved that pentoxifylline inhibits the activation of fibroblasts by pleural effusions ($P < 0.025$) or FCS. This is considered important since both materials, being natural fluids and containing a variety of mediators, probably provide a most appropriate milieu to in vitro study of fibrogenesis.

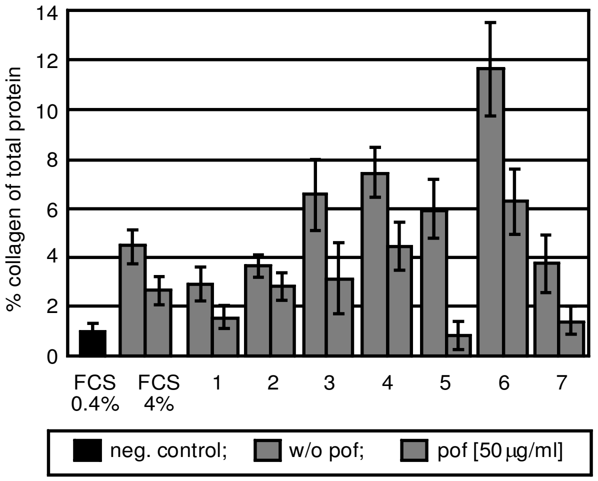

FIG. 5. Effect of pentoxifylline on in vitro fibroblast collagen synthesis, stimulated by fetal calf serum or pleural effusions, with and without addition of pentoxifylline. Fibroblasts (human lung fibroblast cell line WI-38) were investigated at confluent density. Collagen synthesis was determined by comparative measurement of incorporated [$^3$H]-proline and its hydroxylation product [$^3$H]-hydroxyproline and calculated as the collagen share in % of total protein formation. For further details, see legend of Fig. 4. Pentoxifylline inhibited fibroblast activation by pleural effusions ($P < 0.025$). Bars represent means of experiments done in duplicate, error bars the ranges of individual results.

synthesis was calculated per fibroblast. Results did not correlate with concentrations of TGF-β, TNFα, or s-TNF-R.

### Discussion

Transforming growth factor-β (TGF-β) is considered a key cytokine, sustained synthesis of which underlies the development of tissue fibrosis.[11] Screening pleural effusions for several proteins considered to be involved in tissue remodelling (namely tumour necrosis factor α (TNFα), soluble TNF receptor p55 (sTNF-R), and TGF-β), we found TGF-β1 to be positively correlated with development of pleural thickenings in patients with non-carcinomatous pleurisy. Prevention and therapy of fibrosis still being a source of controversy, we searched for alternatives to steroid administration and found evidence that elevating intracellular cAMP, e.g. with pentoxifylline, might be a promising approach.

Our results also highlight the importance of TGF-β in tissue repair. There are three known isoforms of TGF-β in humans (TGF-β 1, 2 and 3) the biological properties of which are nearly identical.[23] TGF-β is strongly chemotactic for fibroblasts and induces these cells to secrete extracellular matrix proteins.[11] It exhibits autoinduction potency[11] and modulates the actions of platelet-derived growth factor, fibroblast growth factor, interleukin-1, and TNFα in such a manner that central role in orchestration of tissue fibrosis can probably be ascribed to it.[23] In an animal model of bleomycin-induced pulmonary fibrosis, neutralizing antibodies to TGF-β isoforms 1 and 2 were able to attenuate the fibrosing processes.[24] The TGF data presented in this study were obtained from ELISA tests on TGF-β. The decision to use ELISA was methodological: bioassays to determine bioactive TGF-β in pleural effusions did not reveal reproducible data.

Since we were looking for pleural fibrosis markers, this turned out to be a crucial decision, since it provided an explanation of why TGF-β might indeed indicate the course of disease: the data includes both active and latent, inactive TGF-β. The physiological function of latent, inactive TGF-β is currently the subject of intensive investigations. It appears likely that not only synthesis of TGF-β, but in particular its activation and deactivation, constitutes a major controlling step in tissue remodelling and that, for instance, activated macrophages might create micro-environments at the site of disease that could contribute to activation of latent TGF-β. High concentrations of latent TGF-β are also known to modulate immune processes.[25] The correlation between TGF-β and pleural thickenings occurring months later opens up perspectives for prevention and therapy of pleural fibrosis: inhibition of TGF-β might be desirable since it is likely that TGF-β is causally involved in fibrogenesis. Further, TGF-β might prove to be a tool for estimating pleural fibrosis risk, although this aspect will have to be addressed in the context of a larger study population.

The poor correlations of TNFα and its soluble receptor sTNF-R p55 with diagnosis or course of disease is not surprising when one considers their brief half-lifes.[26,27] This elucidates two general concerns of *ex vivo* investigations in that the time of sample-drawing after onset of disease is a matter of chance and studying a single probe does not allow for assessment of future developments unless a longitudinal disease marker is used. Although sustained TNF synthesis might be of importance in chronic fibrosis, TNF certainly is no marker for chronic developments, but rather a pro-inflammatory cytokine that is activated by multiple mechanisms other than chronic fibrosing inflammation.

In a search for fibrosis-modulating drugs, we found pentoxifyllin to inhibit *in vitro* human lung fibroblast (WI-38) proliferation and differentiation subsequent to various stimuli as shown in Figs 2–5. The POF concentrations used to cause inhibition are comparable with those used to inhibit *in vitro* TNFα formation: oral or i.v. administration of POF in recommended doses causes a similar reduction in TNF synthesis, as do 50 μg/ml POF in the *in vitro* system.[28]

Pentoxifylline also influences many of the known contributors to fibrogenesis: acute lung injury induced by chemicals and inflammatory mediators is attenuated.[29] Pentoxifylline seems to be protective of pneumocyte function.[30] It inhibits the formation of free radicals effectively[31] as well as formation and action of TNFα,[28] and injury of lungs perfused with human neutrophils.[32] Pentoxifylline inhibits *in vitro* fibroblast proliferation driven by different stimuli (fetal calf serum,[18] platelet-derived growth factor,[19] tumour necrosis factor α[14]), and inhibits synthesis of several fibroblast products like collagen, glycosaminogylcans and fibronectin.[14,18] Pentoxifylline also augments *in vitro* collagenase production by fibroblasts.[18]

Our own data add that (1) TGF-β is most likely a relevant cytokine in pleural fibrosis; (2) pentoxifylline inhibits the effect of transforming growth factor-β on fibrogenesis; (3) the effects, not only of selected cytokines, but even of fluids containing a complex heterogeneity of mediators like pleural effusions, can be inhibited by pentoxifylline in concentrations easily attainable *in vivo*. This is important since, although steroids have been administered in fibrosis for a long time, whether they exert direct inhibitory influence on fibroblasts remains controversial: the *in vitro* data, at least, depend heavily on the culturing methods employed.[33] Thus pentoxifylline may exhibit an advantage in that it inhibits not only inflammatory mediators (as do corticosteroids) but fibrogenesis as well. The inhibition of TGF-β-driven collagen production by pentoxifylline deserves special emphasis since, in a rat model of bleomycin-induced pulmonary inflammation, raised levels of TGF-β1 synthesis by alveolar macrophages was not suppressed by high-dose steroid treatment.[34] This might be one line of explanation for the limited efficacy of steroid treatment in idiopathic pulmonary fibrosis.

In conclusion, we suggest that xanthines—especially pentoxifylline—might be effective in prevention and therapy, not only of pleural fibrosis but of other fibrosing disorders as well. There are many results which seem perfectly suited to the actions of xanthines and the physiopathology of fibrosis. We feel that sufficient evidence now exists to propose pentoxifylline for a prospective therapeutic intervention study in human disease.

### References

1. Crouch E. Pathobiology of pulmonary fibrosis. *Am J Physiol* 1990; **259**: L159–L184.
2. Piguet PF, Ribaux C, Karpuz V, Grau GE, Kapanci Y. Expression and localization of tumor necrosis factor-α and its mRNA in idiopathic pulmonary fibrosis. *Am J Pathol* 1993; **143**: 651–655.
3. Vassalli P, Grau Ge, Piguet PF. TNF in autoimmune diseases, graft-versus-host reactions, and pulmonary fibrosis. *Immunol Ser* 1992; **56**: 409–430.
4. Antoniades HN, Bravo MA, Avila RE. Platelet-derived growth factor in idiopathic pulmonary fibrosis. *J Clin Invest* 1990; **86**: 1055–1064.
5. Broekelmann T, Limper AH, Colby TV, McDonald JA. Transforming growth factor beta 1 is present at sites of extracellular matrix gene expression in human pulmonary fibrosis. *Proc Natl Acad Sci USA* 1991; **88**: 6642–6646.
6. Meier-Sydow J, Weiss SM, Buhl R, Rust M, Raghu G. Idiopathic pulmonary fibrosis: current clinical concepts and challenges in management. *Semin Respir Crit Care Med* 1994; **15**: 77–96.
7. Barret NR. The pleura. With special reference to fibrothorax. *Thorax* 1970; **25**: 515–524.
8. Sahn SA. The pleura. *Am Rev Respir Dis* 1988; **138**: 184–234.
9. Shimokata K, Saka H, Murate T, Hasegawa Y, Hasegawa T. Cytokine content in pleural effusion. *Chest* 1991; **99**: 1103–1107.
10. Barnes PF, Fong SJ, Brennan PJ, Twomey PE, Mazumder A, Modlin RL. Local production of tumor necrosis factor and IFN-gamma in tuberculous pleuritis. *J Immunol* 1990; **145**: 149–154.
11. Border WA, Ruoslahti E. Transforming growth factor-beta in disease: the dark side of tissue repair. *J Clin Invest* 1992; **90**: 1–7.
12. Digel W, Porzsolt F, Schmid M, Herrmann F, Lesslauer W, Brockhaus M. High levels of circulating soluble receptors for tumor necrosis factor in hairy cell leukemia and type B chronic lymphocytic leukemia. *J Clin Invest* 1992; **89**: 1690–1693.
13. Ward A, Clissold SP. Pentoxifylline. *Drugs* 1987; **34**: 50–97.
14. Berman B, Wietzerbin J, Sanceau J, Merlin G, Duncan MR. Pentoxifylline inhibits certain constitutive and tumor necrosis factor-alphainduced activities of human normal dermal fibroblasts. *J Invest Dermatol* 1992; **98**: 706–712.
15. Goldberg ND, Haddox MK, Dunham E, Lopez C, Hadden JW. The yin yang hypothesis of biological control: opposing influences of cyclic GMP and cyclic AMP in the regulation of cell proliferation and other biological processes. In: Clarkson B, Baserga R, eds. *Control of Proliferation in Animal Cells*. New York: Cold Spring Harbor Laboratory, 1974; 609–625.
16. Samlaska MCP, Winfield EA. Pentoxifylline. *J Am Acad Dermatol* 1994; **30**: 603–621.
17. Schade UF. The role of prostacyclin in the protective effects of pentoxifylline and other xanthine derivatives in endotoxin action in mice. *Eicosanoids* 1989; **2**: 183–188.
18. Berman B, Duncan MR. Pentoxifylline inhibits normal human dermal fibroblast in vitro proliferation, collagen, glycosaminoglycan, and fibronectin production, and increases collagenase activity. *J Invest Dermatol* 1989; **92**: 605–610.
19. Peterson TC. Pentoxifylline prevents fibrosis in an animal model and inhibits platelet-derived growth factor-driven proliferation of fibroblasts. *Hepatology* 1993; **17**: 486–493.
20. International Labour Office. *Guidelines for the Use of the ILO International Classification of Radiographs of Pneumoconiosis.* Geneva: Occupational and Health Series No. 22, 1980.
21. Denizot F, Lang R. Rapid colorimetric assay for cell growth and survival. Modifications to the tetrazolium dye procedure giving improved sensitivity and reliability. *J Immunol Methods* 1986; **89**: 271–277.
22. Wiestner M, Krieg T, Hörlein D, Glanville RW, Fietzek P, Müller PK. Inhibiting effect of procollagen peptides on collagen biosynthesis in fibroblast cultures. *J Biol Chem* 1979; **254**: 7016–7023.
23. Border WA, Noble NA. Transforming growth factor β in tissue fibrosis. *N Engl J Med* 1994; **331**: 1286–1292.
24. Giri SN, Hyde DM, Hollinger MA. Effect of antibody to transforming growth factor beta on bleomycin induced accumulation of lung collagen in mice. *Thorax* 1993; **48**: 959–966.
25. Kekow J, Wachsman W, MacCutchan JA, Gross WL, Zachariah M, Carson DA, Lotz M. Transforming growth factor-β and suppression of humoral immune responses in HIV infection. *J Clin Invest* 1991; **87**: 1010–1016.
26. Beutler BA, Milsark IW, Cerami A. Cachectin/tumor necrosis factor: production, distribution, and metabolic fate in vivo. *J Immunol* 1985; **135**: 3972–3977.
27. Lesslauer W, Tabuchi H, Gentz R, Brockhaus M, Schlaeger EJ, Grau G, Piguet PF, Pointaire P, Vassalli P, Loetscher H. Recombinant soluble tumor necrosis factor receptor proteins protect mice from lipopolysaccharide-induced lethality. *Eur J Immunol* 1991; **21**: 2883–2886.
28. Zabel P, Schade FU. Pentoxifylline as an anti-tumour necrosis factor-alpha agent. *Immunol Infect Dis* 1993; **3**: 175–180.
29. Zabel P, Schade FU. Pentoxifylline and tumour necrosis factor-induced lung injury. *Eur Respir J* 1994; **7**: 1389–1391.
30. Balibrea-Cantero JL, Arias-Diaz J, Garcia C, Torres-Melero J, Simon C, Rodriguez JM, Vara E. Effect of pentoxifylline on the inhibition of surfactant synthesis induced by TNF-alpha in human type II pneumocytes. *Am J Respir Crit Care Med* 1994; **149**: 699–706.
31. Ciufetti G, Mercury M, Ott C, Lombardini R, Paltricia R, Lupattelli G, Santambrogio L, Mannarino E. Use of pentoxifylline as an inhibitor of free radical generation in peripheral vascular disease. *Eur J Clin Pharmacol* 1991; **41**: 511–515.
32. McDonald RJ. Pentoxifylline reduces injury to isolated lungs perfused with human neutrophils. *Am Rev Respir Dis* 1991; **144**: 1347–1350.
33. Durant S, Duval D, Homo-Delarche F. Factors involved in the control of fibroblast proliferation by glucocorticoids: a review. *Endocr Rev* 1986; **7**: 254–269.
34. Khalil N, Whitman C, Zuo L, Danielpour D, Greenberg A. Regulation of alveolar macrophage transforming growth factor-beta secretion by corticosteroids in bleomycin-induced pulmonary inflammation in the rat. *J Clin Invest* 1993; **92**: 1812–1818.

ACKNOWLEDGEMENTS. The authors gratefully acknowledge the methodological help in the determination of collagen synthesis by Professor P. K. Müller, University of Lübeck. Authors are deeply indebted to Mrs S. Ross and Mrs S. Kutsch for their expert technical assistance.

**Received 9 October 1996; accepted in revised form 3 December 1996**

